# Embedding Community at the Heart of Psychiatry Education: A Community Hub Placement for Medical Students

**DOI:** 10.1192/bjo.2026.11337

**Published:** 2026-06-30

**Authors:** Natalie Dean, Hayley Lawrence, Katherine Chapman, Miriam Stanyon, Analia Buckley

**Affiliations:** Derbyshire Healthcare NHS Foundation Trust, Derbyshire, United Kingdom

## Abstract

**Aims:**

Undergraduate psychiatry education is traditionally centred on inpatient services and secondary care experiences, despite the majority of mental health support occurring within communities. This imbalance risks reinforcing medicalised understandings of mental ill health and limits student exposure to preventative, psychosocial, and lived-experience led approaches. With evidence that many doctors practise in the regions where they train, there is a clear educational and workforce imperative to embed community engagement early in medical training. This project aimed to develop and evaluate a novel community-based placement at Middle Street Resource Centre, a charitable organisation providing mental health support and wellbeing services, with community, inclusion, and lived experience embedded at the core of medical education.

**Methods:**

Medical students undertook a structured placement within Middle Street Resource Centre, a non-profit, open-access community hub providing psychosocial support without referral. Students embedded within the centre, participating in a range of existing community activities including music sessions, discussion groups, and social café spaces. In addition, the medical education team facilitated structured group sessions on bipolar disorder, mindfulness, and eating disorders which students actively participated in and co-designed. Evaluation was qualitative, drawing on detailed reflective accounts written by students following their placement experience.

**Results:**

Student reflections highlighted profound learning that extended beyond traditional clinical outcomes. Exposure to a non-judgemental, inclusive environment challenged students’ assumptions about mental illness and disability, strengthening empathy and reflective practice. Engagement with community members illustrated the importance of socialconnection, creativity, and belonging in mental wellbeing, and reinforced the preventative role of community services. Students reported increased confidence in communicating sensitively, adapting language to diverse populations, and appreciating the impact of stigma and diagnostic terminology. Observing and learning from staff and volunteers with lived experience further deepened understanding of relational, trust-based support, contrasting with more hierarchical clinical models. Many students described the placement as transformative, shaping how they intended to practise psychiatry and medicine more broadly.

**Conclusion:**

Embedding medical students within community mental health hubs offers powerful educational value and complements traditional psychiatric training. This placement demonstrates a scalable, innovative model that prioritises prevention, inclusion, and lived experience, while fostering empathy, communication skills, and community connection. As we expand such placements across further community hubs, we recognise the potential to strengthen psychiatry education, support workforce sustainability, and place community at the heart of future mental health care.

